# High doses of a national preschool program are associated with the long-term mitigation of adverse outcomes in cognitive development and life satisfaction among children who experience early stunting: a multi-site longitudinal study in Vietnam

**DOI:** 10.3389/fpubh.2023.1087349

**Published:** 2023-12-22

**Authors:** J. A. Robinson, Phuong Thi Thu Dinh

**Affiliations:** ^1^College of Education, Psychology and Social Work, Flinders University, Adelaide, SA, Australia; ^2^Kinder in Wien, Vienna, Austria; ^3^College of Education, Hue University, Hue, Vietnam

**Keywords:** stunting, early childhood education, preschool, low- and middle-income countries, vocabulary, mathematics, reading, life satisfaction

## Abstract

**Background:**

Stunting (low height-for-age) is a marker of cumulative developmental disadvantage that can also contribute to impaired cognitive development and poor psychological wellbeing. Several interventions designed to preserve stunted children’s developmental potential through increasing their cognitive stimulation have proven to be effective. However, their resource-intensive nature limits their sustainability and scalability in the low-and middle-income countries in which 98% of stunted children live. The current study had three aims: to identify the domains of developmental disadvantage associated with stunting at 5 years of age in the Vietnamese context; to examine the relationship between Vietnamese children’s stunting status at 5 years of age, the dose of the national preschool program they received, and their cognitive skills and psychological well-being at 4 ages; and to determine whether some doses of the national preschool program were associated with the mitigation of adverse cognitive and wellbeing outcomes among stunted children.

**Method:**

The *Young Lives Study* in Vietnam (*n* = 2,000; 31 sites) provided archival data that allowed calculation of the approximate dose (in hours) of the preschool program received by children, and longitudinal data on children’s growth (1, 5, 8, 12, and 15 years), receptive vocabulary (5, 8, 12 and 15 years), reading skills, mathematics skills and life satisfaction (each at 8, 12, and 15 years).

**Results:**

Stunting at 5 years of age was associated with diverse aspects of financial and social disadvantage, greater exposure to health risks, lower preventive health care, and constraints on maternal care. Scores for all cognitive variables at all ages were positively associated with preschool dose and negatively associated with stunted growth at 5 years of age. That is, effects associated with stunting and preschool dose at 5 years of age continued to be found during the subsequent 10 years. High doses of preschool education (3,000 h or more) were associated with the mitigation of adverse outcomes for most cognitive variables at most ages.

**Conclusion:**

The current findings raise the possibility that generic preschool programs delivered at high dose may provide a scalable and sustainable intervention to support the life opportunities of children who experience early stunting.

## 1 Introduction

Diverse life circumstances can result in children receiving inadequate nutrition. Recurrent or chronic undernutrition can lead to stunting (linear growth that is more than two standard deviations below the median height of reference children of the same age and gender) (1). In 2020, it was estimated that 146.3 million children under 5 years of age were stunted. Almost all (98%) of these lived in low- and middle-income countries and most lived in Asia (2). In the absence of intervention, the prevalence of stunting typically increases to a peak between 18 months and 5 years of age and then steadily decreases until adolescence [e.g., (3–5)].

Infancy and early childhood are periods in which rapid brain development and the acquisition of foundational skills takes place (6). Consequently, chronic undernutrition during these periods has the potential to cause diverse long-term disruptions to development. For example, infants who are hospitalized for kwashiorkor, a severe form of undernutrition, show structural disruptions in brain development that can be reversed by timely and appropriate nutrition interventions [e.g., (7)]. However, it is not clear that a comparable cause-and-effect relationship exists for community-dwelling children with stunted growth. Children who experience early stunting may show an elevated prevalence of impaired cognitive performance and poorer psychological well-being during early childhood [e.g., (4, 8)], middle childhood [e.g., (9)], and adolescence (10, 11); and a diverse range of poor health, psychological and employment outcomes in adulthood (12, 13). However, whether such disruptions to development are observed appears to be highly sensitive to context and the gender of the child [e.g., (14, 15)]. Most evidence suggests that the relationship between stunting and poor developmental outcomes is not causal (16). Rather, the cumulative developmental disadvantage that contributes to stunting (17) overlaps with the developmental disadvantages that contribute to cognitive delay and poor psychological well-being.

The prevention of stunting requires large-scale structural changes in food, health and social protection systems and the removal of social, cultural and gender barriers to good child nutrition (18). Such changes will be achieved too late for the many millions of children who are currently stunted. Therefore, interventions have been developed to mitigate adverse outcomes for these children. Three broad approaches have been taken. One seeks to improve children’s nutrition via nutrition-specific (e.g., therapeutic foods) or nutrition-sensitive interventions (e.g., water, sanitation, and hygiene programs) (19). Another approach aims to reduce cumulative developmental disadvantage, for example, through unconditional cash transfers [e.g., (20)]. In the third approach, interventions focus on providing stimulating activities and responsive caregiving to support stunted children’s cognitive development and psychological wellbeing [e.g., (9)].

A range of clinic-, centre- and home-based interventions that increase cognitive stimulation are effective in mitigating poor school performance and low intelligence test scores among stunted children [e.g., (21, 22)]. Although children gain the greatest overall benefit when interventions improve both nutrition and cognitive stimulation, cognitive stimulation alone can provide stunted children with long-term protection against a range of adverse cognitive, mental health and employment outcomes [e.g., (13)]. Indeed, one large meta-analysis found that cognitive stimulation led to increases in stunted children’s cognitive and motor skills that were four to five times greater than those achieved by nutrition interventions, even though cognitive stimulation had no effect on children’s height-for-age (23).

However, because most interventions designed to mitigate adverse outcomes for stunted children are resource-intensive, they have limited sustainability and scalability in low- and middle-income countries. The current study examined whether a widely accessible source of cognitive stimulation, existing preschool programs, can also mitigate adverse outcomes for stunted children. Only one previous prospective longitudinal study appears to have explored the association between low height-for-age, attendance at “generic” preschools, and children’s cognitive development. It focused on outcomes for Peruvian infants who, at one year of age, were either stunted or were “at risk” of stunting (defined as having a length-for-age z-score below −1). At 5 years of age, stunted children had higher vocabulary scores if they had attended a formal preschool (*jardin*) for three years than if they had not attended preschool (24). However, there was no evidence that preschool attendance allowed stunted children to “catch-up” to their peers. Instead, the effect size for formal preschool attendance was highest for children who were not stunted or at risk of stunting when they were infants. The study attempted to statistically control for demographic differences that were confounded with whether or not children attended preschool.

Other studies attempt to minimize such confounding while preserving nuances in the data by comparing children who attend preschool but differ in their stunting status and the “dose” of early education they receive [e.g., (25–27)]. Few studies have adopted this design in large, national samples in Asia. One of the exceptions is a cross-sectional study of 3- to 6-year-old children pooled across four countries in East Asia and the Pacific (Cambodia, Mongolia, Papua-New Guinea, and Vanuatu) (28). The results were similar to those reported by Cueto et al. (24). Although stunted children who received higher preschool doses had higher scores for cognitive skills, there was no evidence that preschool dose was associated with a “catch-up” in the performance difference between stunted children and their peers. However, this study measured the duration of preschool attendance in years rather than a more direct and precise measure of preschool dose. It is also unclear whether the positive association between the duration for which stunted children attended preschool and their cognitive performance were transitory or persisted over time. In other contexts, many of the positive effects of early childhood education show rapid “fade-out” after children enter school [e.g., (29)].

Vietnam provides a useful context for comparing the long-term outcomes for stunted children who receive different doses of preschool education because the influence of many confounding variables is minimized. Vietnamese children’s attendance at preschool (Trường Mẫu giáo) is not dependent on their parents holding education-focused aspirations or values, living in an urban location, or their family’s wealth. Between 2003 and 2009, pre-existing very high levels of preschool attendance increased further in the lead-up to one year of preschool education becoming compulsory for all 5-year-old children in Vietnam regardless of their parents’ values or place of residence (Trường Mẫu giáo) (30). To promote equity in education, preschool and school fees are discounted or subsidized for children from poor families and families of war veterans. In addition, there is limited heterogeneity in the content and quality of children’s experiences at preschool and during their subsequent schooling. The Ministry of Education and Training regulates the quality of preschools and schools, sets standards for teacher training, provides the national curriculum, and supervises its implementation (30, 31). However, the dose of preschool education that Vietnamese children receive can differ markedly because they are eligible to begin preschool at any point after their third birthday, and parents can elect to enroll their children in half-day (from 7 am to 12 pm) or full-day (from 7 am to 4–5 pm) programs in public preschools (32) or for longer hours in some private preschools (25). The dose of preschool education children receive appears to depend on many factors (30). In other contexts, the dose–response relationship between preschool attendance and later cognitive performance has been inconsistent [e.g., (33)]. Little is known about this relationship in the unique context of preschool education in Vietnam. The national preschool program also requires that preschools and schools provide meals and snacks to replace those the children would normally receive at home, and provides minimum nutritional standards for these (34). Stunted and non-stunted children receive the same meals. These provide limited support for “catch-up” in growth in the absence of substantial nutritional input from families. For example, for a child attending preschool for 8–9 h per day, meals and snacks are required to provide only 60%–70% of children’s daily energy requirements.

**Figure 1:**
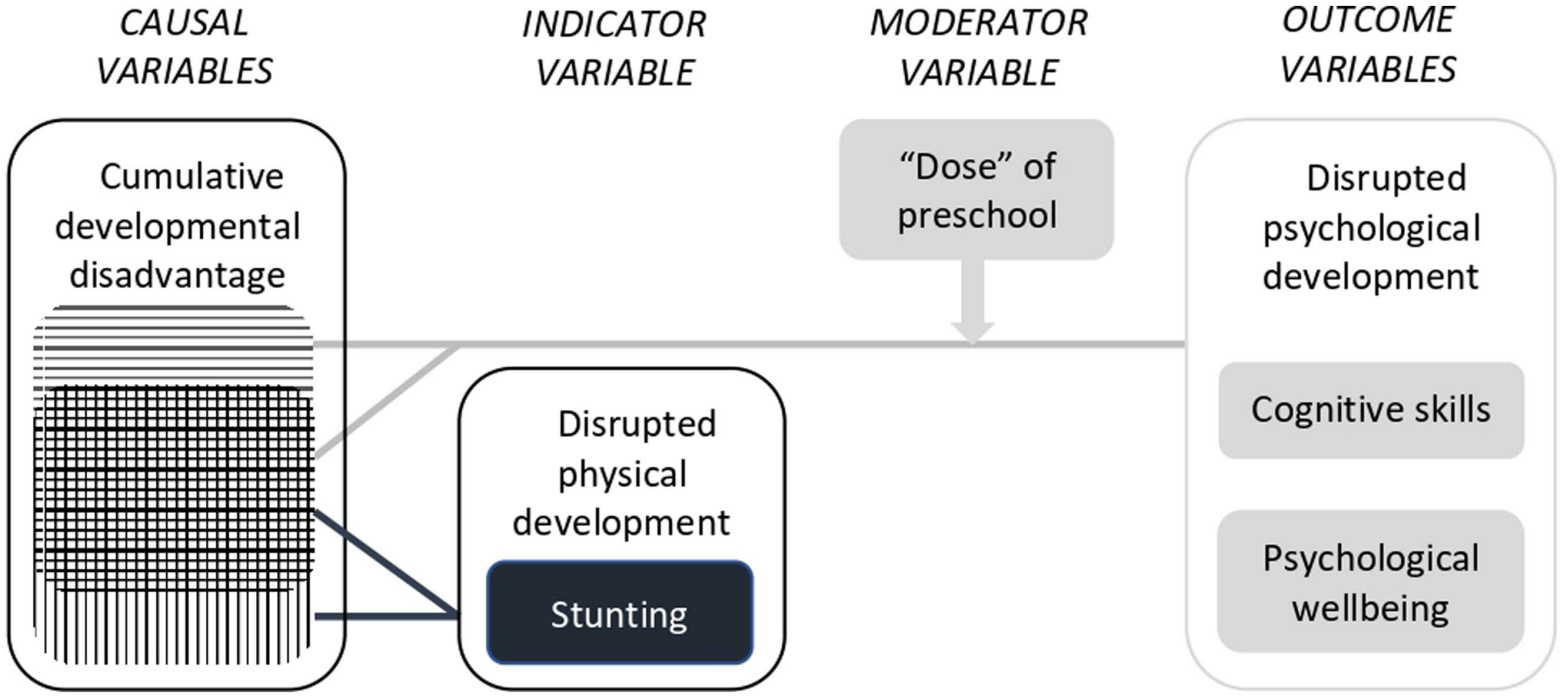
Conceptual model that informed the current study.

The conceptual framework that informed the current study is shown in Figure 1.

This research sought to answer three questions:

1. In the Vietnamese context, which domains of developmental disadvantage are associated with stunting when children are 5 years of age?
2. Are stunting status at 5 years of age and preschool dose associated with differences in Vietnamese children’s
  - Cognitive skills
  - Psychological well-being?

If so, at which ages are differences observed?

1. Are any doses of the national preschool program in Vietnam associated with mitigation of adverse outcomes for children who were stunted at 5 years of age?

If so, for which outcomes and at which ages can this be observed?

## 2 Materials and methods

### 2.1 Sample

This research involved a secondary analysis of archival longitudinal data from the younger Vietnamese cohort in the *Young Lives Study* (35, 36). That research was conducted by the University of Oxford and funded by the UK Department for International Development.

The *Young Lives Study* recruited participants from 31 communes in five provinces across Vietnam: Lao Cai (North-East region), Hung Yen (Red River Delta), Da Nang (Central urban center), Phu Yen (South-Central coast), and Ben Tre (Mekong River Delta). In each province, the study purposefully recruited two of the poorest communes (total of 15 communes; 48% of the sample), one commune of average wealth (total of 9 communes; 29% of the sample) and one commune with above-average wealth (total of 7 communes; 23% of the sample) (37, 38). Due to its focus on children raised in poverty, it recruited no children from communes in the highest wealth quartile (38, 39).

The sample in the current study included all children for whom relevant data were available when they were 1 (*n* = 2,000), 5 (*n* = 1,970), 8 (*n* = 1,961), 12 (*n* = 1,928), and 15 years of age (*n* = 1,912). The attrition rate was very low. At 15 years, 96.8% of the original sample was retained.

The Vietnamese component of the *Young Lives Study* received ethics clearance from the University of Oxford, the Vietnam Union of Science and Technology Association, and the Hanoi School of Public Health (40).

### 2.2 Measures

The timing of data collection is summarized in Table 1.

**Table 1:** Timing of measures.

Measure	Child age				
	1 year	5 years	8 years	12 years	15 years
Height- and weight-for-age	X	X	X	X	X
Receptive vocabulary		X	X	X	X
Reading			X	X	X
Mathematics			X	X	X
Life satisfaction			X	X	X

#### 2.2.1 Growth faltering

To understand stunting at 5 years of age in the broader context of Vietnamese children’s growth, wasting (low weight-for-height) and stunting (low length/height-for-age) were assessed at all data collection points. Children’s length/height and weight were measured in accordance with the WHO’s recommendations (41, 42). At one year of age, infants’ linear growth was measured from the soles of their feet to the top of their head while they were lying flat on their back on a standardized measuring board. At ages 5, 8, 12, and 15 years, children’s standing height without shoes or socks was measured by trained assessors using a portable stadiometer with an accuracy of one millimetre. Children’s date of birth and birthweight data were collected from records made by health clinic staff at the time of the child’s birth (birthweight missing for 11% of children). At all subsequent ages, the child’s weight was measured using scales that were precise to 100 grams. Body mass index (BMI) was calculated as the child’s weight in kilograms divided by the square of their height in metres.

The *WHO Growth Standards* (41) were used to calculate height-for-age and BMI-for-age z scores for children up to 60 months of age. *WHO Growth References* (42) were used to calculate z scores for height-for-age and BMI-for-age *z*-scores at older ages. Z-scores below −5 and above +5 were judged to be implausible. The criterion for stunting was a plausible length/height-for-age z-score under −2. The criterion for wasting was a plausible BMI-for-age z-score under −2.

#### 2.2.2 Dose of the national preschool program

At 5 years of age, primary caregivers answered single items that asked whether, since the age of 3 years, their child had ever attended preschool; the age in months at which they started and stopped attending preschool; the number of hours their child attended per week; the type of preschool they attended (e.g., public, private); whether the child had begun attending school; and the age at which they commenced school.

An approximate measure of preschool dose was calculated as the product of the derived number of months the child attended preschool, the reported hours of attendance per week, and 4 weeks/month. For some analyses, preschool dose was classified into three categories: low (1 to 999 h; approximately equivalent to less than 6 months of full-time attendance), moderate (1,000–2,999 h) and high (3,000 h or more, i.e., approximately equivalent to more than 18 months of full-time attendance).

#### 2.2.3 Cognitive measures

Details about the process of creating and adapting cognitive measures in the Young Lives Study are provided by Dawes (43). The Young Lives team conducted extensive analyses of the reliability and precision of each cognitive test completed by children when they were 5 and 8 years of age. Reliability was assessed using two approaches: Classical Test Theory and Item Response Theory. For the former, a reliability coefficient of 0.60 or more is considered acceptable for research purposes; for the latter, a person-reliability index above 0.50 is considered adequate (44). For Item Response Theory, Rasch modelling was performed on raw data to generate standardised scores. This also allowed any gender bias to be detected in order to estimate and correct for differential item functioning. The precision of measurement was analyzed using the standard error of measurement (SEM) across children. Detailed reports on the results of these analyses are available (44, 45). Key statistics are summarized below.

In the current study, results are reported as Rasch scores where this was feasible. The current authors sought to infer the validity of cognitive measures at each age by examining the correlation between Rasch scores and three socio-demographic variables (household wealth index, and fathers’ and mothers’ education) that typically show small to medium positive correlations with scores on established cognitive tests. At the two youngest ages, the correlation between Rasch scores and children’s age in months was also calculated. Example test items are provided in English translation.

##### 2.2.3.1 Receptive vocabulary

The *Peabody Picture Vocabulary Test-III* (PPVT-III) (46) was designed to assess receptive vocabulary between 2 years 6 months and over 90 years of age. Vocabulary items are arranged in order of difficulty. Testing begins with age-appropriate items, and moves forward or backwards depending on the participants’ performance. The test uses the same a multiple-choice format at all ages. The administrator speaks the word while showing four simple drawings; then asks the participant to point to the picture that best illustrates the word: “Put your finger on the picture that shows ______.” Example vocabulary items that were used in the Young Lives Study when children were 5, 8, 12, and 15 years of age are ‘shoulder’,” “gigantic,” “co-operating” and “dilapidated,” respectively.

A Vietnamese language adaptation of the PPVT-III (44) of was administered at 5 and 8 years of age. Despite its good psychometric properties during pilot testing in Vietnam (44), at 8 years several poorly performing items (i.e., poor item–total correlations and/or bias by gender) and errors in the sequence of difficulty of items were identified. Subsequently, scores at 5 and 8 years were corrected by discarding poorly performing items. At both 5 and 8 years of age, the corrected PPVT showed adequate item reliability using both Classical Test Theory (5 years: Cronbach alpha = 0.96, SEM = 3.6; 8 years: Cronbach alpha = 0.98, SEM = 4.0) and Item Response Theory approaches (5 years: person-reliability index = 0.97, SEM = 17.9; 8 years: person-reliability index = 0.92, SEM = 4.0).

When children with the data required for the analyses in the current study were 5 and 8 years of age, their PPVT-III Rasch scores showed small to moderate positive correlations with their age in months (5 years: *r*_(1409)_ = 0.25; 8 years: *r*_(1599)_ = 0.26), household wealth index (5 years: *r*_(1408)_ = 0.44; 8 years: *r*_(1597)_ = 0.46), and their fathers’ and mothers’ levels of education (Fathers: 5 years: *r*_(1373)_ = 0.29; 8 years: *r*_(1557)_ = 0.41; Mothers: 5 years: *r*_(1400)_ = 0.29; 8 years: *r*_(1584)_ = 0.47).

At subsequent ages, the number of items was reduced and, in consultation with experts in the Vietnamese language, items were reordered to better represent their difficulty in the Vietnamese language (44). The resulting test, which was used at 12 and 15 years, could no longer be described as an adaptation of the PPVT-III but remained a test of receptive vocabulary (47). Therefore, while Rasch scores were used at 5 and 8 years of age; percentage correct scores were reported at 12 and 15 years.

When children with the data required for the analyses in the current study were 12 and 15 years of age, the percentage of correct answers they achieved on the PPVT-III showed small to medium positive correlations with the wealth index for their household (12 years: *r*_(1671)_ = 0.35; 15 years: *r*_(1925)_ = 0.31) and their fathers’ and mothers’ levels of education (Fathers: 12 years: *r*_(1630)_ = 0.35; 15 years: *r*_(1651)_ = 0.30; Mothers: 12 years: *r*_(1660)_ = 0.38; 15 years: *r*_(1679)_ = 0.34).

##### 2.2.3.2 Reading

At 8 years of age children completed three subtests from the *Early Grade Reading Assessment* (EGRA) (48). The subtest on reading familiar words asked the child to read 60 individual words drawn from grade-level texts. Example items include “big,” “walk” and “school.” The subtest for reading and comprehension of passages involved reading aloud a 130-word grade-level text, and after additional time for silent reading, answering eight questions that assessed understanding of the passages’ explicit and inferred meaning. Two sentences from the passage are, “One day, our cat went missing. We thought that she was just playing hide-and-seek, but we could not find he in her favourite places.” The subtest for listening comprehension involved the administrator reading aloud a short passage of text, then asking the child 6 items that assessed understanding of the passages’ explicit and inferred meaning. Two sentences from the passage are, “They both climbed on the log and rowed to shore. ‘We are saved!’ they shouted when they finally arrived on land.” The Ministry of Education and Training, Vietnam has used the Vietnamese language EGRA in the large-scale assessment of early reading abilities (49). In addition, this was the main outcome measure in *Room to Read’s* evaluation of its early literacy intervention in Vietnam (50). In the Young Lives Study, the EGRA showed adequate item reliability using both Classical Test Theory (Cronbach alpha = 0.69) and Item Response Theory (person-reliability index = 0.59) approaches. The standard error of measurement using these approaches was 1.46 and 9.1, respectively.

When children with the data required for the analyses in the current study were 8 years of age, their EGRA Rasch scores were positively correlated with their age in months (*r*_(1639)_ = 0.22) household wealth index (*r* = 0.40), and their fathers’ and mothers’ levels of education (Fathers: *r*_(1595)_ = 0.38; Mothers: *r*_(1625)_ = 0.38).

Custom designed reading tests were administered at 12 (31 items) and 15 years of age (26 items) (51). These were developed based on extensive piloting of a large bank of potential items that assessed letter naming fluency, word fluency, reading fluency, vocabulary knowledge, sentence comprehension, and reading comprehension. Potential items were drawn from commercial tests, national examinations and international reading benchmarking tests, such as the *Progress in International Reading Literacy Study* (PIRLS) (52). For example, at 15 years of age, reading comprehension items were drawn from the *Programme for International Student Assessment* (PISA) and the *United Nations Educational, Scientific and Cultural Organization*’s (UNESCO) *Literacy Assessment and Monitoring Programme* (LAMP) (53). One of the passages of text for the reading comprehension test at 15 years included the following two sentences, “The beauty and power of the jaguar inspired worship among ancient peoples. Possessing a large head and body, the jaguar has legs that are shorter and thicker than a leopard’s.”

When children with the data required for the analyses in the current study were 12 and 15 years of age, the percentage of correct answers they achieved on the age-appropriate reading test showed small to medium positive correlations with the wealth index for their household (12 years: *r*_(1622)_ = 0.37; 15 years: *r*_(1884)_ = 0.27) and their fathers’ and mothers’ levels of education (Fathers: 12 years: *r*_(1585)_ = 0.36; 15 years: *r*_(1616)_ = 0.32; Mothers: 12 years: *r*_(1612)_ = 0.38; 15 years: *r*_(1645)_ = 0.35).

##### 2.2.3.3 Mathematics

At 8, 12 and 15 years of age, custom designed mathematics tests were administered (51). These were developed based on extensive piloting of a large bank of items. Test items increased in difficulty at each age. Rasch scores were used at 8 years of age; percentage correct scores were used at 12 and 15 years.

At 8 years, 9 items assessed basic quantitative and number concepts (counting, knowledge of numbers, number discrimination, and use of basic mathematical operations) and 20 items assessed skills in addition, subtraction, multiplication, and division using whole numbers. Items were presented in both written and spoken forms. Example items that tested the ability to recognise number sequences and complete simple multiplication operations are “24, 26, ___, 30,” and “2 × 4 = __,” respectively. In the Young Lives Study, the Rasch scores for mathematics at 8 years showed adequate item reliability using both Classical Test Theory (Cronbach alpha = 0.90) and Item Response Theory (person-reliability index = 0.88) approaches. The standard error of measurement using these approaches was 1.8 and 4.9, respectively. When children with the data required for the analyses in the current study were 8 years of age, their mathematics Rasch scores showed small to medium positive correlations with their age in months (*r*_(1657)_ = 0.30), household wealth index (*r*_(1655)_ = 0.43) and their fathers’ and mothers’ levels of education (Fathers: *r*_(1613)_ = 0.39; Mothers: *r*_(1643)_ = 0.39).

When the children were 12 (34 items to be completed in 40 min) and 15 years of age (31 items to be completed in 50 min), tests were constructed from questions in national mathematics examinations (Grade 5 and the Certificate of Lower Secondary School), commercial tests, and tests designed to benchmark the mathematics performance of primary and middle school students (*Trends in International Mathematics and Science Study* [TIMMS]) and secondary students (PISA) against an international standard and the performance of students in other nations (43). Vietnam is among the 64 nations that participate in TIMMS (54), and the 92 nations that participate in PISA. The items from these tests used in the *Young Lives Study* assessed number identification, quantity discrimination, pattern identification, calculation, measurement, spatial abilities, and problem solving. An example item assessing calculation at 15 years was, “The mean age of the 11 members of a football team is 22 years. When one member of the football team was sent off, the mean age of the rest of the team was 21 years. How old was the player who was sent off?.” When children in the current study were 12 and 15 years of age, the percentage of correct answers they achieved on the age-appropriate mathematics test showed small to medium positive correlations with the wealth index for their household (12 years: *r*_(1630)_ = 0.41; 15 years: *r*_(1880)_ = 0.29) and their fathers’ and mothers’ levels of education (Fathers: 12 years: *r*_(1590)_ = 0.40; 15 years: *r*_(1615)_ = 0.39; Mothers: 12 years: *r*_(1620)_ = 0.40; 15 years: *r*_(1643)_ = 0.39).

#### 2.2.4 Psychological wellbeing

*Cantril’s Self-anchoring Ladder of Life Satisfaction* (55) is a single-item measure of current global life satisfaction: “Suppose there are nine steps in this ladder, in which the top step of the ladder (score of 9) represents the best possible life and the bottom step (score of 1) represents the worst possible life… Where on the ladder do you feel you personally stand at the present time?”

This measure has proven to be reliable and valid across a range of child and adolescent ages and in diverse cultural contexts [e.g., (56, 57)], including in other samples in Vietnam (58). It has been widely used with school-age children [e.g., (59–61)] and adolescents in diverse low-, middle- and high-income countries [e.g., (62)], including the 51 nations participating in the *Health Behaviour of School-Age Children Study* co-ordinated by WHO [e.g., (63)]. However, it is more useful for examining within-country differences and patterns of change within individuals than for comparing levels of life satisfaction between countries (64). *Cantril’s Ladder* has also been used in studies of adults in Vietnam [e.g., (65)].

## 3 Results

### 3.1 Data analysis

In the conceptual model that informed this study (Figure 1), stunting and impaired cognitive development and psychological wellbeing are all viewed as consequences of multi-dimensional developmental disadvantage. Because stunting is an indicator variable, rather than a causal variable in this model, no attempt was made to covary for the potentially large number of differences in life circumstances, demographic characteristics and growth patterns between children who were and were not stunted at 5 years of age. However, all ANCOVA covaried child age (in months) at the time of assessment. Unless otherwise indicated, effect sizes are reported as partial eta squared ( η _p_^2^).

G*Power 3 for Macintosh (66) was used to calculate the sample size required to detect small effects (Cohen’s d = 0.02) with 80% power when the criterion for significance was *p* < 0.05. The analyses that addressed Research Question 1 sought to identify any aspects of developmental disadvantage that had an elevated prevalence among children who were stunted at 5 years of age. A series of two-group ANCOVA with one covariate compared children who were and were not stunted. For most variables the available sample was more than five times larger than was required (*n* = 244). The analyses that addressed Research Question 2 examined whether differences in children’s outcomes were associated with their stunting status at 5 years and the total number of hours they attended preschool (as continuous data). For a 2-way ANCOVA with one covariate in which both main effects and interactions were of interest, a total sample size of 304 was needed. The available sample for all measures was more than three times this size. Planned comparisons addressing Research Question 3 examined outcomes for children who had and had not been stunted within each preschool dose category to determine whether any dose category mitigated adverse outcomes for children who were stunted at 5 years of age. For most variables, the available sample for these analyses was one-and-a-half to two-and-a-half times larger than the required size (*n* = 244). Because all analyses were overpowered, a criterion of *p* < 0.01 was adopted to minimize the detection of trivial effects and to protect against any Type 1 errors resulting from the large number of analyses needed to answer the research questions.

The main analyses were conducted using SPSS 28.0.1.1 (12).

### 3.2 Prevalence of stunting at 5 years of age

Growth faltering observed at 5 years of age indicated that children’s nutrition was insufficient to support normal growth during the period in which they were eligible to attend preschool or was insufficient to allow catch-up after experiencing undernutrition at earlier ages. At 5 years of age, the prevalence of stunting (25.3%) was high while the prevalence of wasting (3.5%) was low according to the thresholds established by the WHO-UNICEF Technical Expert Advisory Group on Nutrition Surveillance (67). The prevalence of stunting at 5 years of age was eight times higher than the rate in the standardization samples for the *WHO Child Growth Standards* (41) and *Growth Reference Data for 5–19 Years* (42).

Wasting is associated with low energy levels that can impair cognitive performance. However, the prevalence of confounding between stunting and wasting at 5 years of age was very low (*n* = 11). Therefore, wasting was not included as a covariate in analyses for this age group. Because previous research suggests that early wasting is not associated with cognitive delay [e.g., (4)] it was also not necessary to conduct separate analyses for this group, or to include wasting at 5 years of age as a covariate, in analyses at older child ages. Subsequent analyses included all children who were stunted at 5 years of age.

### 3.3 Cumulative developmental disadvantage

Children who were stunted at 5 years of age did not differ from their peers in gender or the mean age at which they were assessed (Table 2). However, they were more likely than their peers to be raised in contexts characterized by diverse forms of developmental disadvantage (Table 2). Stunted children were more likely to be exposed to living conditions (e.g., earthen floor dwelling; unprotected source of drinking water) that increase the risk of parasitic infections and diarrhoeal disease, both of which can directly compromise nutritional sufficiency. In addition, stunted children were more likely than their peers to have elevated nutritional requirements due to infectious disease as result of their housing conditions (e.g., single room dwellings) and lower rates of preventative health interventions (e.g., vaccines). They were also more likely to have mothers with one or more younger children who were also highly dependent on care.

**Table 2:** Comparison between children who were and were not stunted at 5 years of age in life circumstances, demographic characteristics, and growth.

Characteristic	Not stunted (*n* = 1,289)			Stunted (*n* = 437)			Statistic
	*M*	(SD)	%	*M*	(SD)	%	
Male			48.8			54.7	𝜒^2^_(1, *N* = 1726)_ = 4.4
Age (years)							
1 year	12.3	(3.3)		12.2	(3.7)		*t*_(692.8)_ = 0.6^#^
5 years	63.7	(3.6)		63.2	(4.1)		*t*_(682.8)_ = 2.3^#^
8 years	97.2	(3.7)		96.7	(4.1)		*t*_(688.0)_ = 1.3^#^
12 years	146.5	(3.6)		146.1	(4.0)		*t*_(693.0)_ = 1.3^#^
15 years	182.5	(3.6)		182.2	(3.9)		*t*_(690.7)_ = 1.6^#^
Low household capital							
Rural place of residence			77.0			93.6	𝜒^2^_(1, *N* = 1726)_ = 59.1**
Minority ethnic group			5.7			28.6	𝜒^2^_(1, *N* = 1726)_ =207.9**
Parent has no schooling							
Father			4.0			20.1	𝜒^2^_(1, *N* = 1,681)_ = 111.9**
Mother			5.2			28.3	𝜒^2^_(1, *N* = 1726)_ = 174.7**
Parents’ goal for the child is secondary schooling or less			28.9			49.9	𝜒^2^_(1, *N* = 1,667)_ = 61.7**
Poverty at 5 years of age							
Wealth index	0.54	(0.19)		0.40	(0.20)		*t*_(715.2)_ = 12.5^#^**
Household unable to raise 230,000 VND in one week			9.2			16.7	𝜒^2^_(1, *N* = 1722)_ = 18.9**
Health risks							
Unimproved drinking water			62.6			82.3	𝜒^2^_(1, *N* = 1,687)_ = 56.4**
No improved sanitation			12.2			19.3	𝜒^2^_(1, *N* = 1,687)_ = 13.4**
Dwelling has earth floor			16.4			30.0	𝜒^2^_(1, *N* = 1,673)_ = 38.5**
One-room dwelling			38.0			52.4	𝜒^2^_(1, *N* = 1713)_ = 14.0**
Maternal constraints							
Psychologically distressed when child was 1 year old ^			18.2			23.3	𝜒^2^_(1, *N* = 1,625)_ = 4.9
Other babies before the child was 5 years of age			21.9			39.1	𝜒^2^_(1, *N* = 1724)_ = 14.7**
Low preventative health care							
Mother: no antenatal care			20.8			40.4	𝜒^2^_(1, *N* = 1711)_ = 51.9**
Child unvaccinated at 1 year							
No tuberculosis vaccine			9.5			17.4	𝜒^2^_(1, *N* = 1,551)_ = 17.9**
No measles vaccine			30.1			38.2	𝜒^2^_(1, *N* = 1,582)_ = 8.6*
Child growth							
Premature birth (< 37 weeks)			2.8			2.6	𝜒^2^_(1, *N* = 1,691)_ = 0.04
Low birthweight (< 2,500 g)			4.2			6.4	𝜒^2^_(1, *N* = 1,487)_ = 2.6
Stunting at other ages							
1 year of age			7.5			58.0	𝜒^2^_(1, *N* = 1,696)_ = 503.1**
8 years of age			11.7			18.3	𝜒^2^_(1, *N* = 1,652)_ = 11.8**
12 years of age			6.2			56.8	𝜒^2^_(1, *N* = 1726)_ = 541.7**
15 years of age			3.4			38.2	𝜒^2^_(1, *N* = 1,687)_ = 360.1**

^#^Equal variances not assumed. ^*^p < 0.01; ^**^ *p* < 0.001; ns: not significant, *p* > 0.01.^^^Mothers’ symptoms of anxiety and depression during the past 30 days reported on the *Self–Reporting Questionnaire 20* (SRQ–20) (68).

Children who were stunted at 5 years of age were no more likely than their peers to have been born preterm or to have had a low birthweight. However, they had an elevated prevalence of growth faltering as early as 1 year of age, and this continued not only at 5 years but also at all later ages (Table 2).

### 3.4 Preschool attendance

Both stunted children and their peers attended preschool for an average of more than 16 months (Table 3). However, stunted children attended preschool for fewer months and received fewer total hours of the national preschool program than their peers. In addition, although the vast majority of children in both groups attended public preschools, stunted children were less likely than their peers to attend for-profit private preschools (which also deliver the national preschool program).

**Table 3:** Characteristics of preschool attendance by children who were and were not stunted at 5 years of age.

Children who attended preschool	Not stunted at 5 years (*n* = 1,289)			Stunted at 5 years (*n* = 437)			Statistics^
	*M*	(SD)	%	*M*	(SD)	%	
Starting age (months)	43.9	(9.1)		46.7	(8.6)		*t*_(626.0)_ = −5.3**
							*d* = 0.317
Duration of attendance (months)	19.6	(19.6)		16.6	(9.4)		*t*_(610.2)_ = 5.4**
							*d* = 0.317
Preschool dose (hours)	2970.0	(2159.6)		2053.3	(2053.3)		*t*_(718.5)_ = 8.2**
							*d* = 0.442
Preschool dose^#^							𝜒^2^_(2, *N* = 1,552)_ = 54.3**
Low			26.5			36.5	
Moderate			26.3			38.1	
High			47.2			25.4	
Type of preschool							𝜒^2^_(2, *N* = 1,562)_ = 33.5**
Public			84.4			95.6	
Private, for profit			12.1			2.2	
Other (e.g., charity)			3.5			2.2	

^^^*t*-tests were calculated with equal variances not assumed; *d* = Cohen’s d with Hedge’s correction.^**^p < 0.001; ns: not significant, *p* > 0.01.^#^Low: 1 to 999 h; Moderate: 1,000–2,999 h; High: 3,000 h or more.

### 3.5 Differences in children’s outcomes associated with their stunting status at 5 years of age and the dose of the preschool program they received

#### 3.5.1 Cognitive skills

For all domains of cognition at all ages there was a main effect for both children’s stunting status at 5 years of age and the total number of hours they attended preschool (Table 4). Overall, children who were stunted at 5 years of age and children who received lower doses of the preschool program had lower scores on cognitive measures. Preschool dose had a medium effect size for receptive vocabulary at 5 and 8 years of age. All other effect sizes for preschool dose, and all effect sizes for stunting status, were small. However, the effect size for preschool dose was more than twice as large as that for stunting status for most cognitive variables (receptive vocabulary scores at all four ages, reading skills at 12 years, and mathematics skills at 12 and 15 years). An interaction between stunting status at 5 years and preschool dose was found for only one variable at one age (receptive vocabulary at 12 years of age). The effect size was small.

**Table 4:** Differences in outcomes associated with children’s stunting status at 5 years and their total hours of preschool attendance: a summary of results of ANCOVA covarying for child age at the time of assessment.

Measure and child age	Stunting status at 5 years of age			Dose of national preschool program			Interaction: stunting status × preschool dose		
	Statistic	Sig	$\eta_{p}2$	Statistic	Sig	$\eta_{p}2$	Statistic	Sig	$\eta_{p}2$
Receptive vocabulary									
5 years	*F*_(1,1,483)_ = 23.3	**	0.020	F_(2,1,483)_ = 50.7	**	0.083	*F*_(2,1,483)_ = 1.0	ns	–
8 years	*F*_(1,1,439)_ = 25.3	**	0.017	*F*_(2,1,439)_ = 60.6	**	0.078	*F*_(2,1,439)_ = 2.7	ns	–
12 years	*F*_(1,1,128)_ = 46.8	**	0.010	*F*_(2,1,128)_ = 30.6	**	0.040	*F*_(2,1,128)_ = 7.4	**	0.010
15 years	*F*_(1,1,271)_ = 9.1	*	0.002	*F*_(2,1,271)_ = 14.7	**	0.023	*F*_(2,1,271)_ = 3.9	ns	–
Reading									
8 years	*F*_(1,1,513)_ = 43.3	**	0.028	*F*_(2,1,513)_ = 19.9	**	0.026	*F*_(2,1,513)_ = 0.6	ns	–
12 years	*F*_(1,1,446)_ = 19.5	**	0.013	*F*_(2, 1,446)_ = 28.5	**	0.038	*F*_(2,1,446)_ = 2.8	ns	–
15 years	*F*_(1,1,492)_ = 32.2	**	0.021	*F*_(2,1,492)_ = 16.6	**	0.022	*F*_(2,1,492)_ = 1.7	ns	–
Mathematics									
8 years	*F*_(1,1,646)_ = 45.0	**	0.029	*F*_(2,1,646)_ = 19.8	**	0.026	*F*_(2,1,646)_ = 0.3	ns	–
12 years	*F*_(1,1,451)_ = 23.6	**	0.016	*F*_(2,1,451)_ = 36.2	**	0.048	*F*_(2,1,451)_ = 2.0	ns	–
15 years	*F*_(1,1,490)_ = 25.1	**	0.017	*F*_(2,1,490)_ = 32.4	**	0.042	*F*_(2,1,490)_ = 0.4	ns	–
Life satisfaction									
8 years	*F*_(1,1,512)_ = 11.4	**	0.007	*F*_(2,1,512)_ = 2.0	ns	–	*F*_(2,1,512)_ = 1.3	ns	–
12 years	*F*_(1,1,483)_ = 6.8	*	0.005	*F*_(2,1,483)_ = 8.4	**	0.011	*F*_(2,1,483)_ = 4.8	*	0.006
15 years	*F*_(1,1,511)_ = 8.1	*	0.005	*F*_(2,1,511)_ = 11.5	**	0.015	*F*_(2,1,511)_ = 1.8	ns	–

^*^p < 0.01. ^**^p < 0.001; ns: not significant, *p* > 0.01.

#### 3.5.2 Life satisfaction

At all ages, children’s ratings of their life satisfaction showed a main effect for stunting status at 5 years of age (Table 4). The effect size was very small at all ages. At two ages there was also moderate (12 years) and large (15 years) main effects for the total number of hours children attended preschool. Overall, children who had been stunted at 5 years of age and children who received lower doses of preschool reported lower levels of life satisfaction. Mirroring the results for cognitive variables, an interaction between stunting status and preschool dose was found only at 12 years of age. The size of the interaction effect was very small.

### 3.6 Association between preschool dose categories and mitigation of adverse outcomes for children with early stunting

#### 3.6.1 Cognitive skills

**Figure 2:**
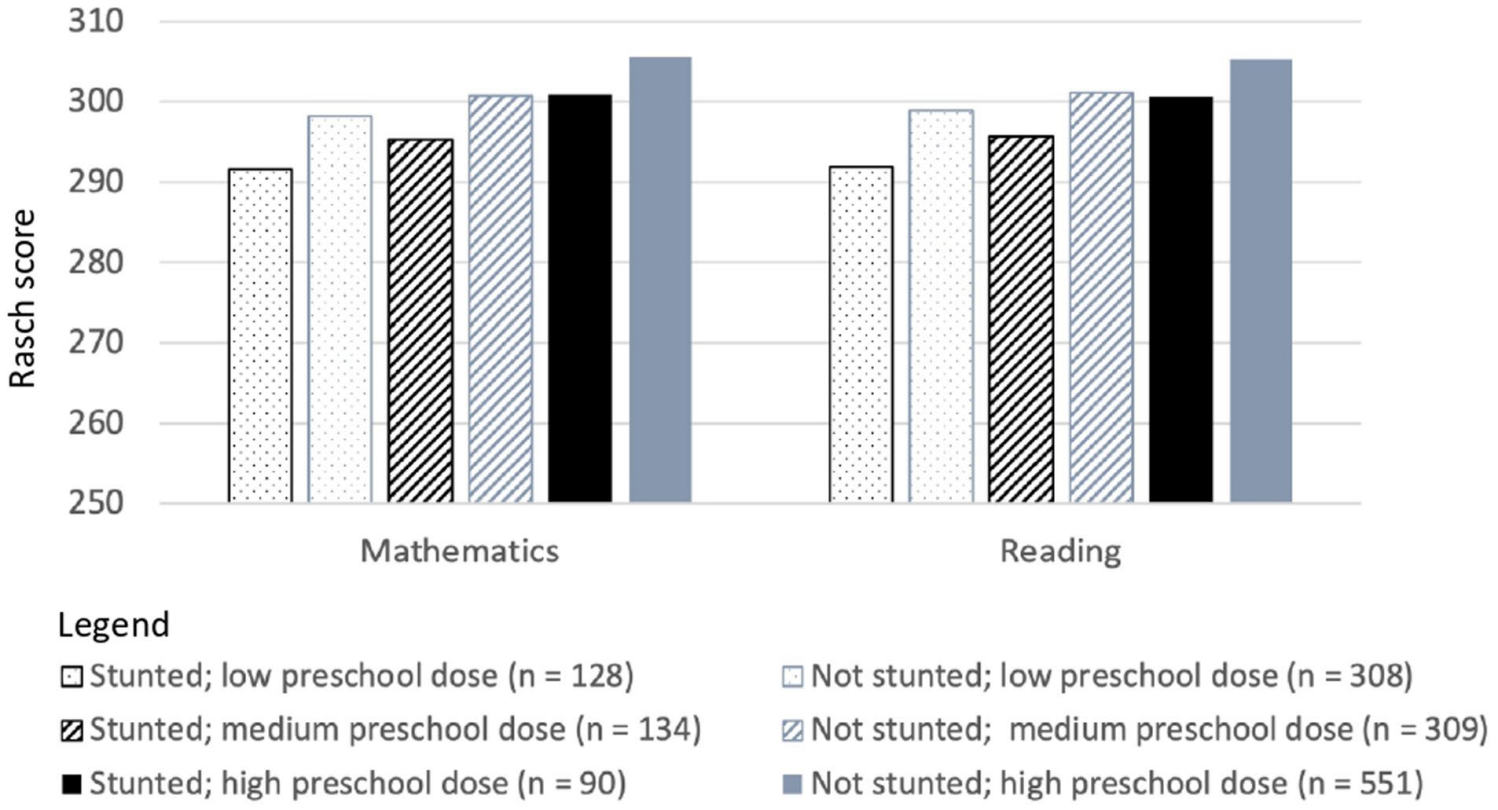
Single time-point Rasch scores for mathematics and reading skills at 8 years of age for Vietnamese children who differed in stunting status at 5 years of age and in the dose of the national preschool program they received.

**Figure 3:**
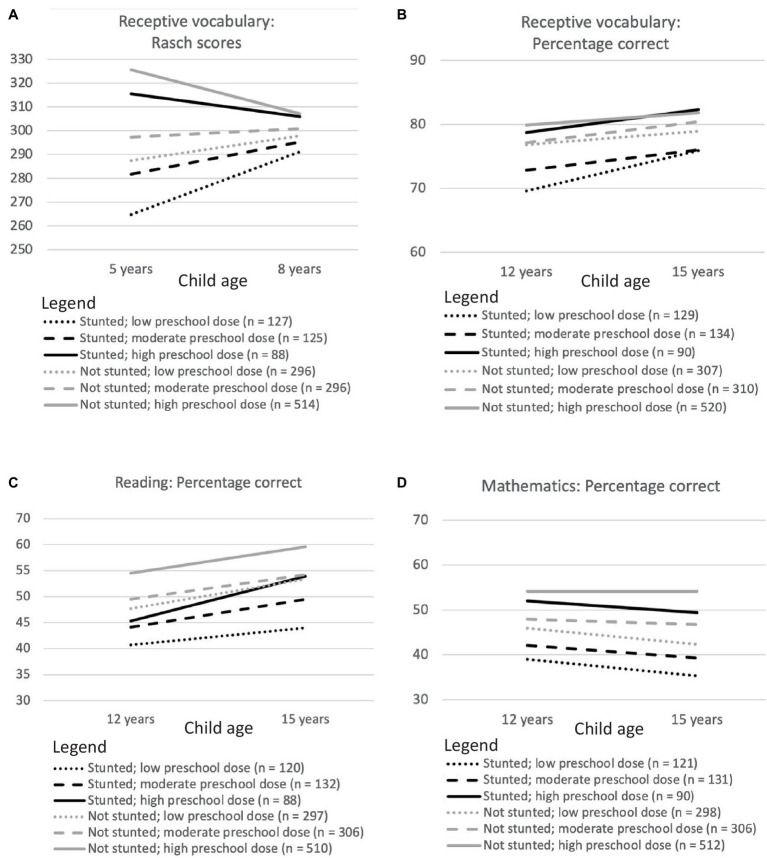
Longitudinal changes in Rasch scores for receptive vocabulary between 5 and 8 years of age **(A)** and in percentage correct scores for receptive vocabulary **(B)**, reading skills **(C)** and mathematics **(D)** between 12 and 15 years of age for Vietnamese children who differed in stunting status at 5 years of age and in the dose of the national preschool program they received.

The results of planned comparisons are summarized in Figures 2, 3 and Table 5.

**Table 5:** Differences in outcomes between Vietnamese children who were and were not stunted at 5 years of age: a summary of planned comparisons between children in the same preschool dose category^.

	Dose of preschool								
	Low (1–999 h)			Moderate (1,000–2,999 h)			High (3,000 h or more)		
Measure and child age	Statistic	Sig	$\eta_{p}2$	Statistic	Sig	$\eta_{p}2$	Statistic	Sig	$\eta_{p}2$
Receptive vocabulary									
5 years	*F*_(1,343)_ = 12.4	**	0.035	*F*_(1,391)_ = 9.3	*	0.036	*F*_(1,573)_ = 4.2	ns	–
8 years	*F*_(1,420)_ = 16.5	**	0.038	*F*_(1,418)_ = 16.7	**	0.038	*F*_(1,599)_ = 0.7	ns	–
12 years	*F*_(1,433)_ = 34.5	**	0.074	*F*_(1,441)_ = 15.2	**	0.033	*F*_(1,607)_ = 2.0	ns	–
15 years	F_(1,436)_ = 20.6	**	0.045	*F*_(1,442)_ = 11.0	**	0.024	*F*_(1,631)_ = 0.6	ns	–
Reading									
8 years	F_(1,433)_ = 24.8	**	0.054	*F*_(1,440)_ = 15.4	**	0.034	*F*_(1,638)_ = 7.7	**	0.012
12 years	*F*_(1,414)_ = 16.3	**	0.038	*F*_(1,435)_ = 9.9	*	0.022	*F*_(1,595)_ = 0.3	ns	–
15 years	*F*_(1,424)_ = 22.7	**	0.051	F_(1,440)_ = 6.3	ns	–	*F*_(1,626)_ = 6.9	*	0.011
Mathematics									
8 years	*F*_(1,427)_ = 23.9	**	0.053	*F*_(1,436)_ = 18.1	**	0.040	*F*_(1,637)_ = 8.8	*	0.014
12 years	*F*_(1,416)_ = 17.5	**	0.040	*F*_(1,434)_ = 11.8	**	0.027	*F*_(1,599)_ = 1.1	ns	–
15 years	*F*_(1,423)_ = 11.1	**	0.026	*F*_(1,439)_ = 12.7	**	0.028	*F*_(1,626)_ = 3.8	ns	–
Life satisfaction									
8 years	*F*_(1,434)_ = 9.3	*	0.021	*F*_(1,437)_ = 4.6	ns	–	*F*_(1,639)_ = 0.5	ns	–
12 years	*F*_(1,433)_ = 13.9	**	0.031	F_(1,441)_ = 2.4	ns	–	F_(1,607)_ = 0.5	ns	–
15 years	*F*_(1,436)_ = 8.2	*	0.018	*F*_(1,443)_ = 3.3	ns	–	*F*_(1,629)_ = 0	ns	–

^^^ANCOVA covarying for child age in months at the time of assessment. ^*^p < 0.01. ^**^p < 0.001; ns: not significant, *p* > 0.01.

##### 3.6.1.1 Receptive vocabulary

When they received only a small or moderate dose of the preschool program, children who had stunted growth at 5 years of age had more limited receptive vocabularies than their peers at every age (Figures 3A,B). However, all differences had a small effect size. At high doses of the preschool program, there was no difference at any age in the size of receptive vocabularies among children who had and had not been stunted at 5 years.

##### 3.6.1.2 Reading

At low doses of the preschool program, children who had been stunted at 5 years of age had lower reading scores than their peers at every age (Figures 2, 3C). At moderate and high doses of the preschool program, there was mitigation of adverse outcomes in reading at one age (at 15 years for children who received a moderate preschool dose; at 12 years for children who received a high preschool dose). The effect size for all differences was small.

##### 3.6.1.3 Mathematics

When they received low or moderate doses of the preschool program, children who had been stunted at 5 years of age had lower mathematics scores than their peers at every age (Figures 2, 3D). In contrast, when children received a high dose of the preschool program, there was no difference at 12 or 15 years age in the mathematics skills of children who were and were not stunted at 5 years of age. The effect size for all differences was small.

#### 3.6.2 Life satisfaction

**Figure 4:**
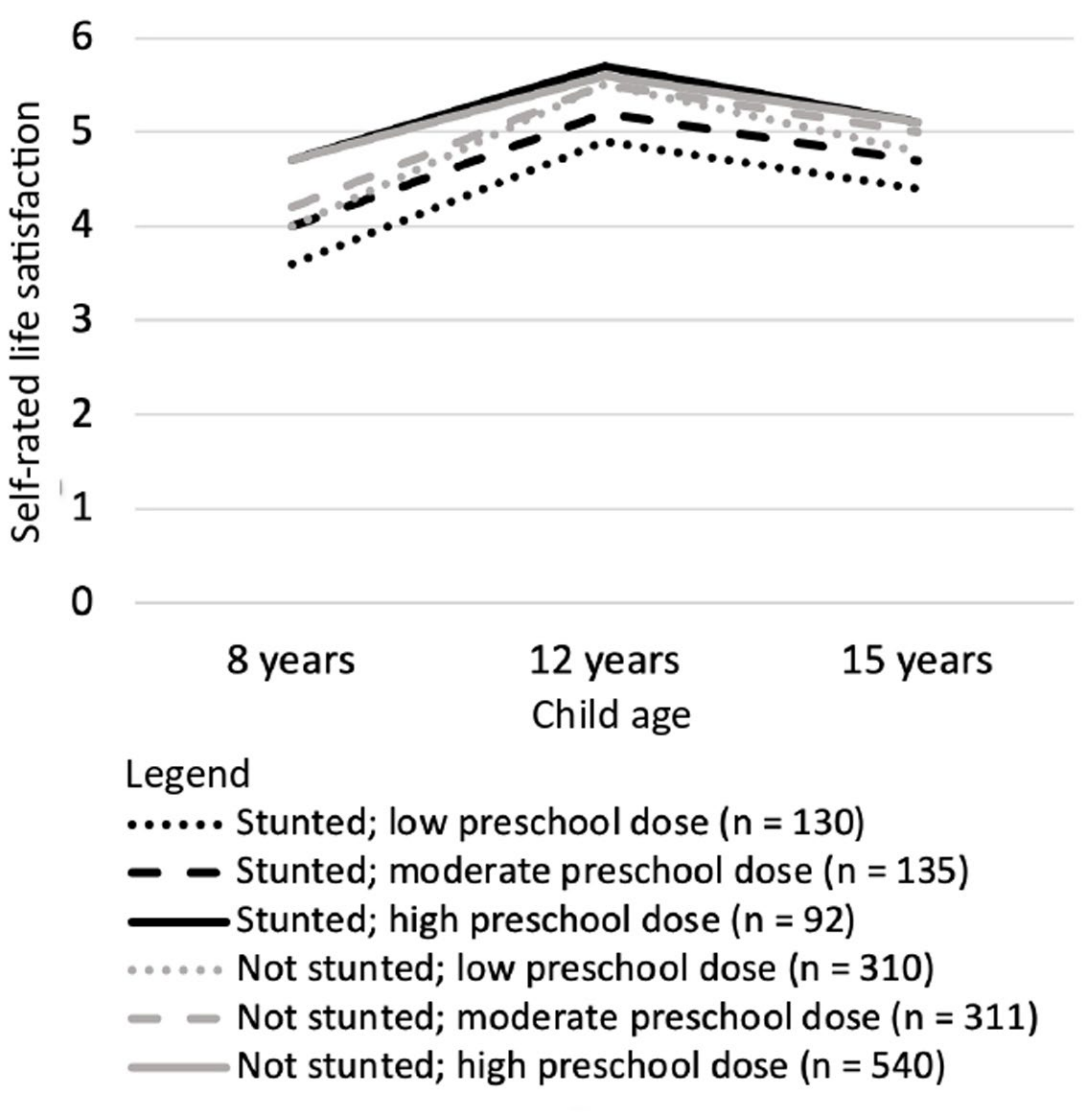
Longitudinal changes between 8 and 15 years of age in life satisfaction reported by Vietnamese children who differed in stunting status at 5 years of age and in the dose of the national preschool program they received.

When children received only a low dose of the preschool program, those who had been stunted at 5 years of age reported lower life satisfaction than their peers at every age (Table 5). However, the effect size for all differences was small. For moderate and high doses of the preschool program, there was no difference at any age in the life satisfaction reported by children who and were not stunted at 5 years (see Figure 4).

### 3.7 Post-hoc analyses

**Figure 5:**
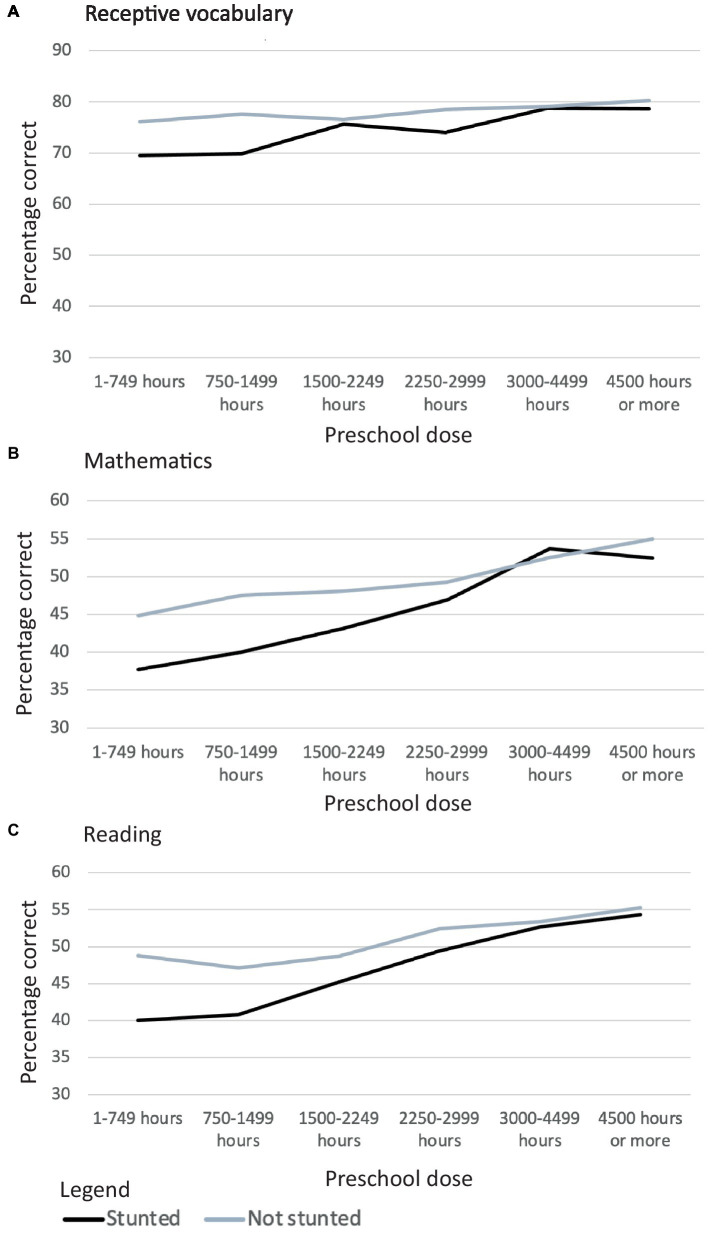
Relationship between Vietnamese children’s stunting status at 5 years of age, the dose of preschool they received*, and their scores in three domains of cognition, receptive vocabulary **(A)**, Mathematics **(B)**, and Reading **(C)** when they were 12 years of age. * 500-hour categories, except when a 3000-3999 category was required to ensure *n* > 20.

Typically, “catch-up” by stunted children would be detected both by interaction terms when a variable was analyzed as continuous data and in planned analyses when the variable was analyzed in ordinal categories. When preschool dose was analyzed as a continuous variable no interactions between it and stunting status were detected for most outcome variables. However, planned analyses showed mitigation of adverse effects when children received high doses of the preschool program. Such a discrepancy would occur if the rate of catch-up was too low to be detected in the linear analysis of continuous data or if there was a non-linear threshold relationship between preschool dose and “catch-up.” To explore these possibilities, finer divisions of preschool dose were examined by visual inspection. Figure 5 shows “catch-up” at 12 years: the only child age at which the mitigation of adverse effects was found for all three cognitive domains, and the only age in which an interaction between preschool dose and stunting status was found for a cognitive variable. There is little evidence of a threshold effect. Instead, the most plausible explanation appears to be that a catch-up of less that 10 percentage points across 3,000 h of preschool represented a very small difference in the slope of the lines for children who were and were not stunted at 5 years of age. Consequently, linear analyses detected this difference only for reading.

In addition, two sets of post-hoc analyses examined whether preschool dose categories for stunted children were confounded with key variables, and if so, whether this could account for the mitigation of adverse effects at high preschool doses identified by the planned comparisons and confirmed by Figure 5. To parallel the planned comparisons, ANCOVA were based on the three dose categories. Due to the relatively small sample size for children who were stunted at 5 years of age, a criterion of *p* < 0.05 was applied in all post-hoc analyses.

The first series of analyses compared the prevalence of prior and subsequent growth faltering across the three preschool dose categories for children who were stunted at 5 years. There was no difference in the prevalence of low birthweight, or stunting at 1, 8 or 15 years of age. However, there was a small difference at 12 years of age (Supplementary Table S1). Follow-up analyses showed that children who received a high preschool dose were less likely to be stunted at 12 years than children who received either a moderate or low dose (high versus moderate: χ ^2^_(1, *N* = 230)_ = 10.5, *p* < 0.001, *r* = 0.21; high versus low: χ ^2^_(1, *N* = 224)_ = 6.3, *p* < 0.05, *r* = 0.17; moderate versus low: χ ^2^_(1, *N* = 270)_ = 0.6, ns). However, this confounding is unlikely to account for the relationship between preschool dose and children’s outcomes at 12 years of age. The three preschool dose categories continued to differ for every outcome variable even after both child age and stunting status at 12 years of age were entered as co-variates (receptive vocabulary: *F*_(2, 350)_ = 8.4, *p* < 0.001, η _p_^2^ = 0.046; mathematics: *F*_(2, 339)_ = 11.0, *p* < 0.001, η _p_^2^ = 0.061; reading: *F*_(2, 337)_ = 12.0, *p* < 0.001, η _p_^2^ = 0.067; life satisfaction: *F*_(2, 349)_ = 6.9, *p* < 0.001, η _p_^2^ = 0.038).

The second series of analyses examined demographic variables. Despite policies to promote equity in preschool education in Vietnam, preschool dose was confounded with urban/rural location ( χ ^2^_(2, *N* = 362)_ = 13.9, *p* < 0.001), and with household wealth at all ages at which it was possible to assess this: 1, 5, and 15 years of age (Table 6). Wealth differences between preschool dose categories predated children entering preschool and were persistent. However, this confounding cannot account for dose-dependent differences in cognitive outcomes for stunted children. Even after both urban/rural location and wealth were added to child age as co-variates, the three preschool dose categories continued to differ in receptive vocabulary at every age, and in both reading and mathematics skills at 12 and 15 years of age (Table 7).

**Table 6:** Differences in household wealth between preschool dose categories for children who were stunted at 5 years of age: results of ANCOVA covarying for child age.

	Dose of preschool received by children who were stunted at 5 years of age								
	Low		Moderate		High				
Wealth index	Mean	(SD)	Mean	(SD)	Mean	(SD)	Statistic	Sig	$\eta_{p}2$
1 year	0.279	(0.186)	0.332	(0.190)	0.480	(0.143)	*F*_(2,358)_ = 27.8	**	0.137
5 years	0.365	(0.196)	0.407	(0.182)	0.532	(0.143)	F_(2,358)_ = 18.4	**	0.093
15 years	0.610	(0.154)	0.651	(0.150)	0.747	(0.084)	*F*_(2,353)_ = 20.7	**	0.105

^**^p < 0.001.

**Table 7:** Differences in outcomes associated with preschool dose categories for children who were stunted at 5 years of age: results of post-hoc ANCOVA covarying for child age, household wealth index and urban/rural location.

	Dose of preschool received by children who were stunted at 5 years of age								
	Low		Moderate		High				
Measure and child age	Mean	(SD)	Mean	(SD)	Mean	(SD)	Statistic	Sig	$\eta_{p}2$
Receptive vocabulary									
5 years	265.8	(35.5)	280.2	(47.6)	314.3	(45.1)	*F*_(2,277)_ = 17.8	***	0.114
8 years	291.1	(13.3)	295.2	(15.6)	305.9	(13.5)	*F*_(2,334)_ = 9.1	***	0.052
12 years	69.6	(15.6)	72.9	(13.1)	78.7	(8.5)	*F*_(2,349)_ = 4.8	**	0.027
15 years	71.4	(14.1)	75.1	(13.3)	82.7	(10.5)	*F*_(2,350)_ = 9.9	***	0.054
Reading									
8 years	291.3	(17.8)	295.0	(15.4)	300.0	(12.0)	*F*_(2,335)_ = 0.2	ns	–
12 years	40.7	(16.5)	44.2	(17.3)	53.7	(16.9)	*F*_(2,336)_ = 6.0	**	0.035
15 years	44.0	(17.3)	49.5	(17.8)	53.9	(17.3)	*F*_(2,340)_ = 3.6	*	0.021
Mathematics									
8 years	291.6	(13.5)	295.3	(14.9)	300.9	(13.8)	*F*_(2,337)_ = 1.2	ns	–
12 years	39.0	(16.0)	42.4	(17.8)	52.3	(18.1)	*F*_(2,339)_ = 5.6	**	0.032
15 years	35.3	(18.0)	39.3	(19.5)	49.4	(22.6)	*F*_(2,338)_ = 3.1	*	0.018
Life satisfaction									
8 years	5.5	(2.3)	5.5	(2.1)	6.0	(2.1)	*F*_(2,344)_ = 0.1	ns	–
12 years	4.9	(1.5)	5.2	(1.7)	5.7	(1.4)	*F*_(2,348)_ = 9.1	***	0.050
15 years	5.6	(1.9)	5.7	(1.6)	6.0	(1.4)	*F*_(2,349)_ = 0.3	ns	–

^*^p < 0.05. **p < 0.01. *p* < 0.001; ns: non-significant, *p* > 0.05.

The interpretation of findings for life satisfaction is less clear. The planned comparisons found mitigation of adverse outcomes in life satisfaction when stunted children received moderate or high doses of the preschool program. In contrast, when preschool dose was analyzed as a continuous variable, it showed a main effect for life satisfaction only at 12 years of age. After both urban/rural location and household wealth were added as covariates to the post-hoc analyses, the three preschool dose categories also differed in life satisfaction only at 12 years of age (Table 7). However, it is unlikely that the mitigation of adverse outcomes in life satisfaction is an artefact of confounding between preschool dose and rural/urban location. Location accounted for less than 0.2% of the variance in life satisfaction at all ages (8 years: *r*_(417)_ = −0.044, ns; 12 years: *r*_(422)_ = −0.001, ns; 15 years: *r*_(424)_ = −0.015, ns). Similarly, the plausibility of household wealth serving as a determinant of children’s life satisfaction is limited by the instability of the relationship between these variables across child age (8 years: *r*_(417)_ = 0.364, *p* < 0.001; 12 years: *r*_(422)_ = 0.080, ns; 15 years: *r*_(424)_ = 0.239, *p* < 0.001) and within preschool dose categories, where the relationship between wealth and life satisfaction was sometimes negative (12 years, high preschool dose: *r*_(91)_ = −0.243, *p* < 0.05).

## 4 Discussion

### 4.1 Developmental disadvantage

Stunting reflects cumulative developmental disadvantage sufficient to compromise physical growth (17). Vietnamese children who were stunted at 5 years of age were more likely than their peers to experience fifteen of the sixteen dimensions of developmental disadvantage that were assessed in this study. These included proxy measures of diverse aspects of financial and social capital, exposure to health risks, low preventive health care, and constraints on maternal care. Children who were stunted at 5 years of age were also more likely than their peers to be stunted at 8, 12 and 15 years of age.

### 4.2 Relationship between preschool dose as a continuous measure, stunting status at 5 years, and children’s outcomes

Stunting during early childhood was associated with concurrent and future impaired cognitive development and psychological wellbeing. Overall, children who were stunted at 5 years had smaller vocabularies, poorer reading and mathematics skills and lower life satisfaction at every age. However, differences between stunted children and their peers were small. When preschool dose was analyzed as a continuous variable (total hours of attendance), higher doses were associated with larger vocabularies, better reading and mathematics skills and higher life satisfaction at every age. Thus, there was no evidence that the positive association between preschool dose and stunted children’s cognitive skills or life satisfaction “faded” over time. These positive associations had a longevity that continued until the age at which many children in low and middle-income countries complete their formal education. An interaction between preschool dose and stunting status was found for only one cognitive variable at one age, and for life satisfaction at one age. Both these effects were small. Thus, similar positive relationships between preschool dose and outcomes were seen for children who were and were not stunted at 5 years of age. However, the failure to find interactions suggested that children who were stunted at 5 years of age did not “catch-up” to their peers in either cognitive development or psychological wellbeing.

### 4.3 Association between preschool dose and mitigation of adverse outcomes

A different picture about “catch-up” emerged when children who were stunted at 5 years of age were compared with peers in the same preschool dose category. There was no evidence that low doses of preschool were associated with mitigation of adverse outcomes for stunted children on any measure at any age. Moderate doses of preschool were primarily associated with mitigation of adverse outcomes in life satisfaction. In contrast, high doses of preschool were associated with mitigation of adverse outcomes in life satisfaction at all ages, receptive vocabulary at all ages, mathematics skills at two ages, and reading skills at one age. Moreover, even in some cases in which children who had been stunted at 5 years of age had lower cognitive scores than their peers in the high preschool dose category, their scores were comparable to those of children who were not stunted but received only a moderate dose of preschool education. The discrepancy between the linear analyses and the planned comparisons concerning “catch-up” is attributable to the small difference between stunted children and their peers in the slope of the relationship between preschool dose and all outcomes. Each point in the narrowing of the disadvantage experienced by stunted children “cost” many additional hours of preschool.

### 4.4 Results in context

The current results confirm previous findings that children with early stunting show persistent impaired cognitive development [e.g., (4, 8–10)], and that the dose of preschool they receive is positively associated with their cognitive skills (24, 28). Unlike many other studies [e.g., (29)], this research found no evidence of “fade-out” in the association between preschool dose and cognitive outcomes.

Like previous research using less fine-grained measures of preschool dose, the current study found little evidence of an interaction between stunting status and preschool dose when it was analyzed as a continuous variable (28). However, this study appears to be the first to find evidence that, at high doses, preschool attendance is associated with the long-term mitigation of adverse outcomes on diverse domains of cognition. When they received high doses of preschool, children who were stunted at 5 years of age “caught-up” to their peers in most of the cognitive variables that were assessed in this study. This finding initially appears to be inconsistent with Cueto et al.’s (24) finding that lengthy attendance at formal preschools did not lead to catch-up in receptive vocabulary for Peruvian children who experienced, or were at risk of experiencing, stunting at one year of age. However, it seems likely that the children in that study would have been assigned to the moderate preschool dose category in the current study. Peruvian *jardin* offer only a half-day program. The current study also found no evidence of catch-up in the size of stunted children’s receptive vocabularies at 5 years of age when they received a moderate dose of formal preschool.

The current study is the first to report a very consistent association between preschool dose and subsequent life satisfaction among children who were stunted at 5 years of age. Moderate and high doses of preschool were associated with the mitigation of adverse outcomes for life-satisfaction at all the ages at which this was assessed. However, further research is needed for a clear interpretation of this finding.

### 4.5 Mechanisms

The current study’s observational research design precludes conclusions about cause and effect. It is plausible that relationships between preschool dose, cognition and life satisfaction observed among both stunted and non-stunted children are an artefact of confounding between preschool dose and causal variable(s). Post-hoc analyses suggest that it is unlikely that these relationships can be accounted for by differences between preschool dose categories in prior or subsequent growth faltering, household wealth or urban/rural location. However, it remains plausible that children’s outcomes were influenced by confounding between preschool dose and variables that were not analyzed in the current study.

It is also plausible that the relationship between preschool dose and cognitive development is attributable to one or more components of the preschool program. In Vietnam, the national preschool program provides cognitive stimulation through play and the academic curriculum; access to water, sanitation, and hygiene (WASH) facilities that may not be available in children’s homes; and meals and snacks. The latter two components are nutrition-sensitive and nutrition-specific factors, respectively. However, it is unlikely that nutrition-related factors are responsible for the relationship between preschool dose and cognitive outcomes. Any improvement in children’s nutrition resulting from WASH facilities or the provision of food in preschools was insufficient to overcome undernutrition. Children who experienced stunting at age 5 had, on average, attended preschool for 16 months and received access to WASH facilities as well as meals and snacks during that time, before being identified as stunted. Moreover, previous research suggests that even when nutrition interventions are effective in overcoming stunting during early childhood, they provide few benefits for cognitive development unless they are integrated with interventions that improve cognitive stimulation or responsive caregiving [e.g., (10, 22, 23, 69)]. Such integrated interventions often yield similar improvements in cognitive development to interventions that focus exclusively on stimulation or caregiving [e.g., (21, 22)]. In addition, nutrition-related factors cannot explain why the relationship between preschool dose and cognitive outcomes did not differ between children who were and were not stunted at 5 years of age. When preschool dose was analyzed as a continuous variable, no interaction between stunting status and dose was found in nine out of ten analyses, and the effect size in the one exception was small.

In the absence of evidence to the contrary, the most plausible explanation for the current pattern of findings is that diverse domains of cognitive development are supported by the formal and informal learning activities provided by preschools in Vietnam. Extensive previous research has demonstrated that interventions that directly or indirectly increase stunted children’s cognitive stimulation are effective in improving diverse domains of cognition in both the short- and long-term [e.g., (13, 22, 70)].

### 4.6 Limitations

The current study has several limitations that should be considered when interpreting its findings and drawing policy implications. First, the study used an observational research design, which precludes the identification of cause-and-effect relationships. The prospective longitudinal nature of the design excludes some directions of effect. It is not feasible that later cognitive and well-being outcomes influenced early stunting or preschool dose. However, it is possible that one or more unexamined factors that are confounded with high preschool doses account for the mitigation of the adverse effects of early stunting observed in this study. This limitation is unavoidable in studies seeking to answer the current research questions. It is not possible to conduct experimental research on the effects of a standardized national preschool education program. However, this limitation warrants serious consideration when drawing policy implications from such studies. Second, if subsequent research confirms that the positive outcomes documented in the current research are due to the cognitive stimulation provided by the preschool program, the current research is unable to provide insights into the formal and informal learning activities that were most effective. In addition, there was no comparison with outcomes from other methods of improving cognitive stimulation for stunted children that may have lower cost. Interventions that deliver cognitive stimulation through community groups or home visiting programs [e.g., (71, 72)] have proven to be effective and may also deliver benefits for other children in the household.

### 4.7 Directions for future research

One useful direction for future research would be to examine dynamic relationships within and between domains of development for stunted children. Such research might identify processes that are self-productive (73). That is, where a high level of skill at early ages facilitates rapid subsequent gains in skills in the same domain. One consequence of self-productive processes is that over time children with poor initial levels of skill may fall further and further behind their peers unless they receive an effective intervention. In the current study, the gap in reading skills between stunted children who received low and high doses of preschool increased over time (Figure 3). Other processes in cognitive development may show cross-productivity. For example, if stunted children’s receptive vocabulary is expanded by preschool attendance (or other factors), they may be better able to benefit from mathematics and reading instruction when they enter school. Developmental processes may also show evidence of dynamic complementarity over time (73). This occurs when early investments in development raise the productivity of investments at subsequent ages but are also most (or only) effective if these follow-up investments are made. Although the current findings concerning high preschool doses are consistent with dynamic complementarity, the study did not collect the data necessary to test it. Research investigating these issues could both inform the design and timing of interventions and contribute to the development of theory.

### 4.8 Policy implications

Caution needs to be exercised when drawing policy implications from the current findings. In addition to the limitations outlined above, the context of preschool education in Vietnam is unique among low- and middle-income countries. Vietnamese education is heavily influenced both its one-party communist form of government (30) and its Confucian cultural heritage, which ascribes a high value to education (74). It also has an unusually long history of preschool education, which has resulted in well-established infrastructure and culturally informed curricula and teacher training programs (31). These factors have led to a national preschool program with distinctive characteristics. These were an asset in the current research because they minimized the effect of many variables that are confounded with preschool dose in other contexts. However, these same characteristics limit the policy implications of the current findings for other low- and middle-income countries because the effectiveness of policies and interventions is influenced by their sensitivity to local resources, environments, political processes, and cultures.

In addition, like interventions to overcome the cumulative developmental disadvantage that contributes to stunting and poor cognitive development and wellbeing, preschool programs are a significant long-term expense. Policymakers in low- and middle-income countries make difficult decisions about the most efficient use of limited funding. Vietnam needed to make sacrifices in other areas to achieve universal preschool education. Between 2008 and 2013, during the lead up and first five years of mandatory preschool education, Vietnam dedicated between 17 and 19 percent of all government expenditure to education (75). Many high-income countries allocated a smaller percentage to education during this period (75), even though they were not also attempting to address critical shortfalls in basic health services, nutrition, and public infrastructure. Vietnam continues to dedicate a large percentage of the national budget to education (14.8 percent in 2020). Many other low- and middle-income countries are unlikely to be able to match this sustained commitment.

However, there currently appears to be a window of opportunity for the establishment and expansion of preschool programs. A range of international agencies have recently advocated for, and provided funding towards, universal preschool programs in low- and middle-income countries [e.g., (76–78)]. Several of these countries are now implementing (or plan to implement) two- or three-year formal preschool programs, which have the potential to match the high doses of preschool associated with positive outcomes in the current study (77). Despite the limits on their generalizability, the current findings suggest that there is a possibility that such initiatives may have the unexpected secondary benefit of mitigating adverse cognitive outcomes among children who experience cumulative developmental disadvantage that leads to early stunting. If this proves to be the case, there may be substantial benefits for both the financial and personal well-being of millions of individuals and for national economic productivity and development.

### 4.9 Conclusion

Global rates of stunting during childhood have shown a sustained decrease over recent decades (2, 79). Despite this, the prevention of stunting remains a “wicked problem” (80) in many low- and middle-income countries. In the current study, stunting status was associated with fifteen diverse aspects of developmental disadvantage. It is unlikely that such disadvantages will be overcome quickly. Therefore, strategies that are effective in preserving the developmental potential of children who experience early stunting are likely to be needed for the foreseeable future. The current findings raise the possibility that, in addition to their other benefits, generic preschool education programs delivered at high doses may provide a scalable and sustainable intervention to support the life opportunities of these children.

## Data availability statement

Publicly available datasets were analyzed in this study. This data can be obtained by application to The Young Lives study at the University of Oxford https://www.younglives.org.uk/data.

## Ethics statement

The studies involving humans were approved by Young Lives has subsequently received approval before each pilot and round of data collection from the ethics committees at the University of Oxford and each one of the study countries, as follows: – Central University Research Ethics Committee (CUREC), Social Science Division, University of Oxford (since 2005) – Hanoi School of Public Health Research (since 2015). The studies were conducted in accordance with the local legislation and institutional requirements. Written informed consent for participation in this study was provided by the participants’ legal guardians/next of kin.

## Author contributions

JR and PD contributed to all sections of the manuscript. All authors contributed to the article and approved the submitted version.
