# Supplementary material for: High doses of a national preschool program are associated with the long-term mitigation of adverse outcomes in cognitive development and life satisfaction among children who experience early stunting: a multi-site longitudinal study in Vietnam
Source: Front Public Health. 2023 Dec 22;11:1087349. doi: 10.3389/fpubh.2023.1087349 (PMC10770864; doi:10.3389/fpubh.2023.1087349)
Supplement: Supplementary file 1 [file Table_1.docx]

**Supplementary tables**

Supplementary Table 1. Differences in prior and subsequent growth faltering associated with three categories of preschool dose for children who were stunted at 5 years of age

|  | Prevalence among children who were stunted at 5 years of age (%) | | | | |  |  |  |
| --- | --- | --- | --- | --- | --- | --- | --- | --- |
|  | Dose of preschool education | | | | |  | |  |
| Growth faltering | Low |  | Moderate |  | High |  | Statistic | Sig |
| Low birthweight^ | 6.8 |  | 5.0 |  | 4.6 |  | $\chi$^2^_(2, N=275)_ = 0.5 | ns |
| Stunting |  |  |  |  |  |  |  |  |
| 1 year | 53.5 |  | 65.6 |  | 54.5 |  | $\chi$^2^_(2, N=346)_ = 4.6 | ns |
| 8 years | 24.6 |  | 17.0 |  | 13.5 |  | $\chi$^2^_(2, N=350)_ = 4.7 | ns |
| 12 years | 58.3 |  | 63.0 |  | 41.3 |  | $\chi$^2^_(2, N=362)_ = 11.1 | * |
| 15 years | 42.3 |  | 33.3 |  | 32.6 |  | $\chi$^2^_(2, N=357)_ = 3.1 | ns |

^ Under 2,500 g

* p <.01; ns: not significant, p > .05
